# Multi-domain knowledge graph embeddings for gene-disease association prediction

**DOI:** 10.1186/s13326-023-00291-x

**Published:** 2023-08-14

**Authors:** Susana Nunes, Rita T. Sousa, Catia Pesquita

**Affiliations:** LASIGE, Faculdade de Ciências, Universidade de Lisboa, Lisboa, Portugal

**Keywords:** Ontologies, Knowledge graph, Knowledge graph embeddings, Machine learning, Gene-disease association prediction

## Abstract

**Background:**

Predicting gene-disease associations typically requires exploring diverse sources of information as well as sophisticated computational approaches. Knowledge graph embeddings can help tackle these challenges by creating representations of genes and diseases based on the scientific knowledge described in ontologies, which can then be explored by machine learning algorithms. However, state-of-the-art knowledge graph embeddings are produced over a single ontology or multiple but disconnected ones, ignoring the impact that considering multiple interconnected domains can have on complex tasks such as gene-disease association prediction.

**Results:**

We propose a novel approach to predict gene-disease associations using rich semantic representations based on knowledge graph embeddings over multiple ontologies linked by logical definitions and compound ontology mappings. The experiments showed that considering richer knowledge graphs significantly improves gene-disease prediction and that different knowledge graph embeddings methods benefit more from distinct types of semantic richness.

**Conclusions:**

This work demonstrated the potential for knowledge graph embeddings across multiple and interconnected biomedical ontologies to support gene-disease prediction. It also paved the way for considering other ontologies or tackling other tasks where multiple perspectives over the data can be beneficial. All software and data are freely available.

**Supplementary Information:**

The online version contains supplementary material available at 10.1186/s13326-023-00291-x.

## Introduction

More than 1,400 Mendelian conditions (single genetic locus) present an unknown molecular cause [1]. Additionally, almost all medical conditions are somehow influenced by human genetic variation. Despite the advancements in genomics over the past two decades, identifying the genetic basis of diseases remains an open challenge. Furthermore, most diseases present a highly heterogeneous genotype, which hinders biological marker identification. Diseases like Autism Spectrum Disorder that often have multiple etiologies with the involvement of possibly hundreds of different genes represent an additional challenge [2]. However, this challenge also presents itself as an opportunity to understand the mechanisms of diseases and human biology by exploring the interplay between genes, phenotypes, and diseases, uncovering new diagnostic markers, and therapeutic targets.

Genomic studies and high-throughput experiments, such as linkage studies, generate a large amount of data that can point toward associations between genes and diseases. However, precisely validating these associations in the wet lab is expensive and time-consuming. This fostered the development of computational approaches for predicting gene-disease associations and identifying the most promising associations to be further validated. These approaches typically explore diverse databases (e.g., DisGeNet [3], dbSNP [4]) and employ various computational approaches ranging from machine learning to network-based algorithms.

Opap and Mulder [5] have identified three main challenges in gene-disease associations:

1. how to represent the data in a readily accessible manner for researchers;
2. how to attribute evidence to assertions made by algorithms;
3. how to scale the algorithms with the rate of increase in data size and complexity.

Methods that explore the scientific knowledge described in ontologies can provide an answer to the first two challenges. Ontologies are formal and explicit specifications of a conceptualization of a given domain [6]. They provide a structured way to define concepts and relations between them and have been used in the biomedical domain for the past two decades to support a shared and computationally amenable description of biological entities. Hundreds of biomedical ontologies have been developed, covering almost all domains of biological and biomedical research. Biomedical ontologies have become increasingly important to structure and describe existing biological knowledge and have propelled a new panorama of semantic biomedical data, where millions of semantically described biomedical entities are annotated with ontology concepts and structured in knowledge graphs. Knowledge graphs structure and link data described through an ontology, creating a graphical representation of the information [7]. However, ontologies and knowledge graphs do not directly address the third challenge, but can be explored by different algorithmic approaches to tackle the challenges of data size and, perhaps more importantly, complexity.

**Fig. 1:**
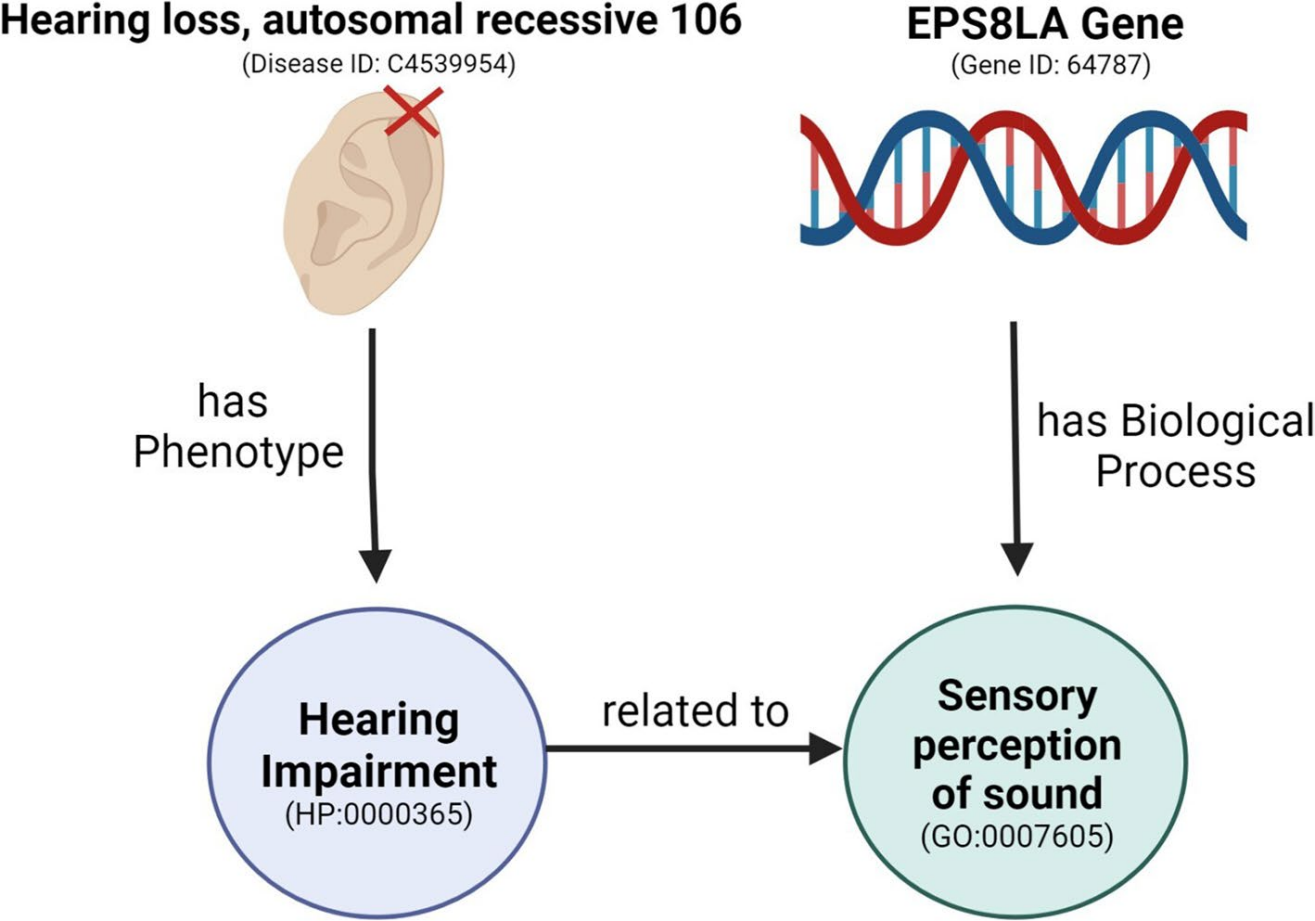
Example of a direct relationship between hearing loss and the EPS8LA Gene

Several well-established works explore biomedical ontologies to support gene-disease association prediction, with some exploring ontology annotations directly [8, 9] while others use semantic similarity [2, 10, 11], i.e. the similarity between two entities based on their shared meaning under an ontology [12]. More recently, approaches based on knowledge graph embeddings (KGE) [13] have also been successful in predicting gene-disease associations [14–16]. Knowledge graph embeddings allow the representation of each entity with a vector that approximates the similarity properties of the graph and can then be used either to compute similarity or to feed a machine learning algorithm. Knowledge graph embeddings support in principal more powerful representations than semantic similarity since they consider multiple types of relations and are multi-dimensional. However, these works employ straightforward approaches that work either over a single ontology [14, 16] or multiple but disconnected ones [15] ignoring potential semantic links across different ontologies. In a complex task such as predicting gene-disease associations, employing a single ontology may be insufficient since multiple perspectives, such as gene function and phenotype, may be necessary for prediction, and establishing richer connections between the ontologies can help integrate the different perspectives. Figure 1 shows an example of a relationship between a gene and disease through gene functions and phenotypes. In preliminary work, we established that knowledge graph embeddings outperform semantic similarity measures in gene-disease prediction and that combining multiple ontologies has the potential to support gene-disease prediction [17].

The main goal of this work is to investigate the impact of the semantic richness of the knowledge graph in the prediction of gene-disease associations employing knowledge graph embeddings. Our guiding hypothesis is that richer representations covering both multiple domains and linking them with more complex relations can improve the performance of knowledge graph embeddings methods in gene-disease association prediction. We propose a novel approach for gene-disease prediction that is based on building rich knowledge graphs to represent both genes and diseases under multiple richly connected ontologies and then exploring it with existing knowledge graph embeddings methods. We investigate the role of logical definition and compound ontology mappings in establishing links between different ontologies and how different knowledge graph embeddings methods effectively explore them.

## Methods

### Overview

We model the problem of predicting gene-disease associations as a supervised learning task where positive examples are pairs of one gene and one disease related to it, and negative examples are pairs of genes and diseases without known association. Genes and diseases are represented by vectors generated by applying knowledge graph embeddings methods over a knowledge graph composed of genes, diseases and ontologies that describe them. These embeddings are combined with different strategies to represent gene-disease pairs, which are then fed to machine learning models for training.

**Fig. 2:**
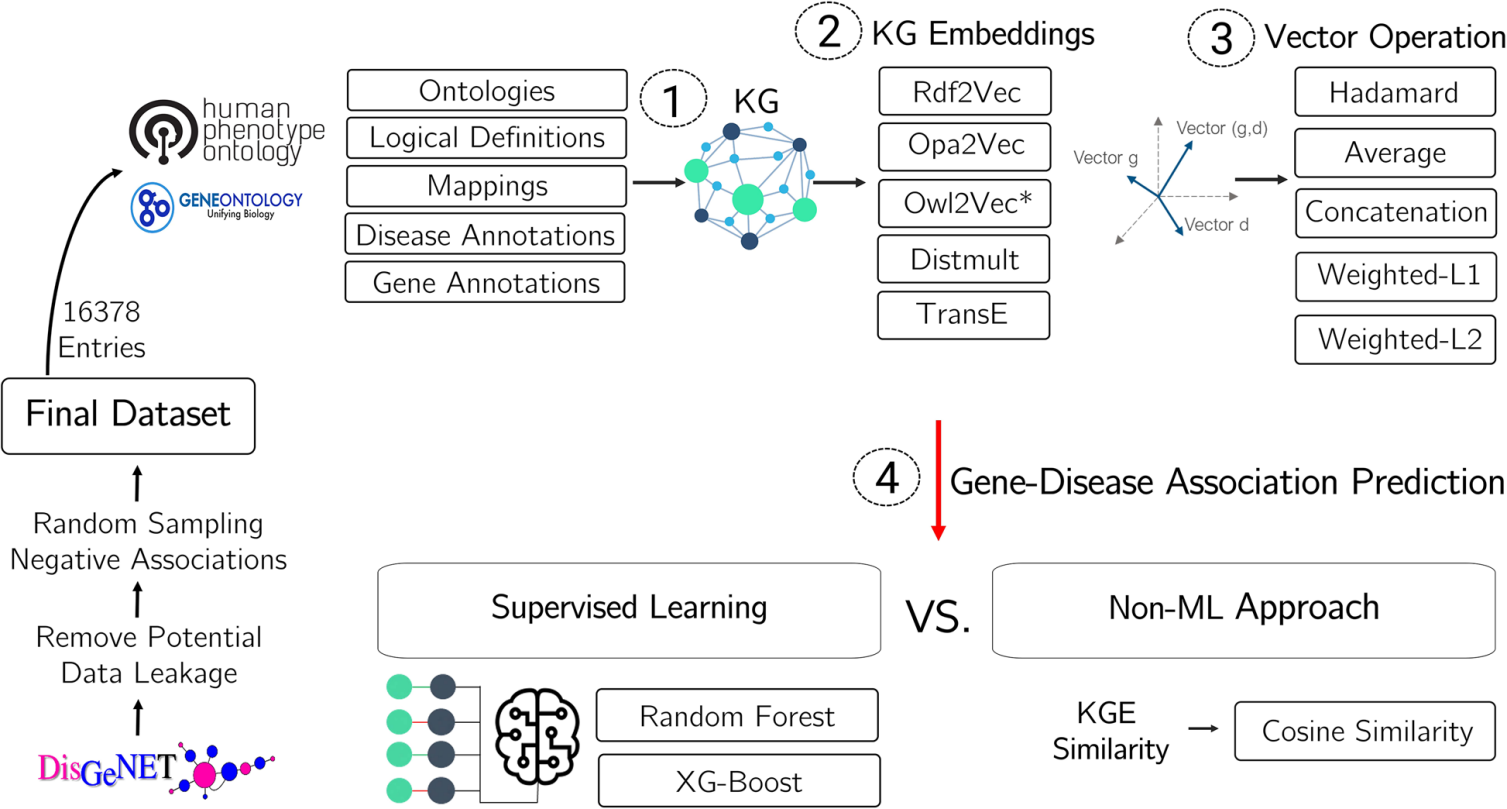
Overview of the methodology with four basic steps: 1) build the knowledge graph with ontologies and annotations; 2) create embeddings to represent each gene and disease; 3) produce a final vector of the pairs in the dataset; 4) gene-disease association prediction

An overview of the methodology is shown in Fig. 2. In a preliminary step, we create the gene-disease association dataset by exploring DisGeNET. Then, the first step in the approach is to integrate the different ontologies and annotation data to build the knowledge graph. In a second step, the embeddings that represent the gene and the disease according to their representation in the knowledge graph are created. In a third step, these embeddings are combined using vector operators producing a representation of genes and diseases in what then becomes a shared semantic space. Finally, in a fourth step, supervised learning algorithms are trained over the embeddings to predict gene-disease associations. This approach is also evaluated against a non-machine learning-based baseline that computed the cosine similarity of knowledge graph embeddings.

This methodology is applied to a diverse set of knowledge graphs that we create to evaluate the impact of the semantic richness of the knowledge graph on gene-disease prediction. All experiments were performed on the same machine^1^.

### Data

#### Gene-disease associations

84038 curated gene-disease associations were extracted from DisGeNET - a discovery platform that contains a comprehensive catalogue of genes and variants associated with human diseases [3]. These pairs corresponds to combinations between 9703 genes and 11181 diseases, with the average number of genes per disease and diseases per gene being two. These pairs were then filtered to account for potential data leakage. Data leakage is when information from outside the training dataset is used to create the model [18]. DisGeNET [3] includes gene-disease associations extracted from multiple sources, including Uniprot [19], OMIM [1], or Orphanet [20], which are the same sources used to create some of the ontology annotations. These gene-disease pairs were filtered out resulting a total of 73469 pairs, composed of 8545 genes and 6490 diseases remained. Finally, only genes and diseases with annotations to HP and GO, or just HP respectively, were kept. This resulted in a total of 2716 genes, 1807 diseases, and 8189 gene-disease associations.

Considering that negative samples are not included in DisGeNET, we employed a random sampling method to create negative examples composed of the genes and diseases present in the positive examples but without known associations between them, building a final balanced dataset with 16378 entries.

#### Ontologies and knowledge graphs

The knowledge graphs built to support the experiments are composed of one (or more ontologies) and the gene and disease annotations to them. We selected the Human Phenotype Ontology (HP) since it provides annotations of both genes and diseases according to the phenotypes they are related to and the Gene Ontology (GO), that provides functional annotations for gene products.

The Human Phenotype Ontology provides a comprehensive resource for the analysis of human diseases and phenotypes, offering a computational bridge between genome biology and clinical medicine. This ontology is organized as five independent subontologies covering different categories: Frequency, Clinical Course, Clinical Modifier, Mode of Inheritance and Phenotypic Abnormality [21, 22]. In addition, it also provides annotations to diseases and human genes. In the latter case, all phenotype classes linked to a disease caused by variants of a certain gene are assigned to that gene.

The Gene Ontology is the most successful case of the use of an ontology in biomedical research, supporting the functional annotation of gene products for multiple species under three branches: biological process, molecular function and cellular component [23, 24]. The Gene Ontology Annotation initiative provides annotations for gene products, which associate a gene product with a GO class (also referred to as a GO term) identified the type of evidence behind the association.

#### Logical definitions and ontology mappings

**Fig. 3:**
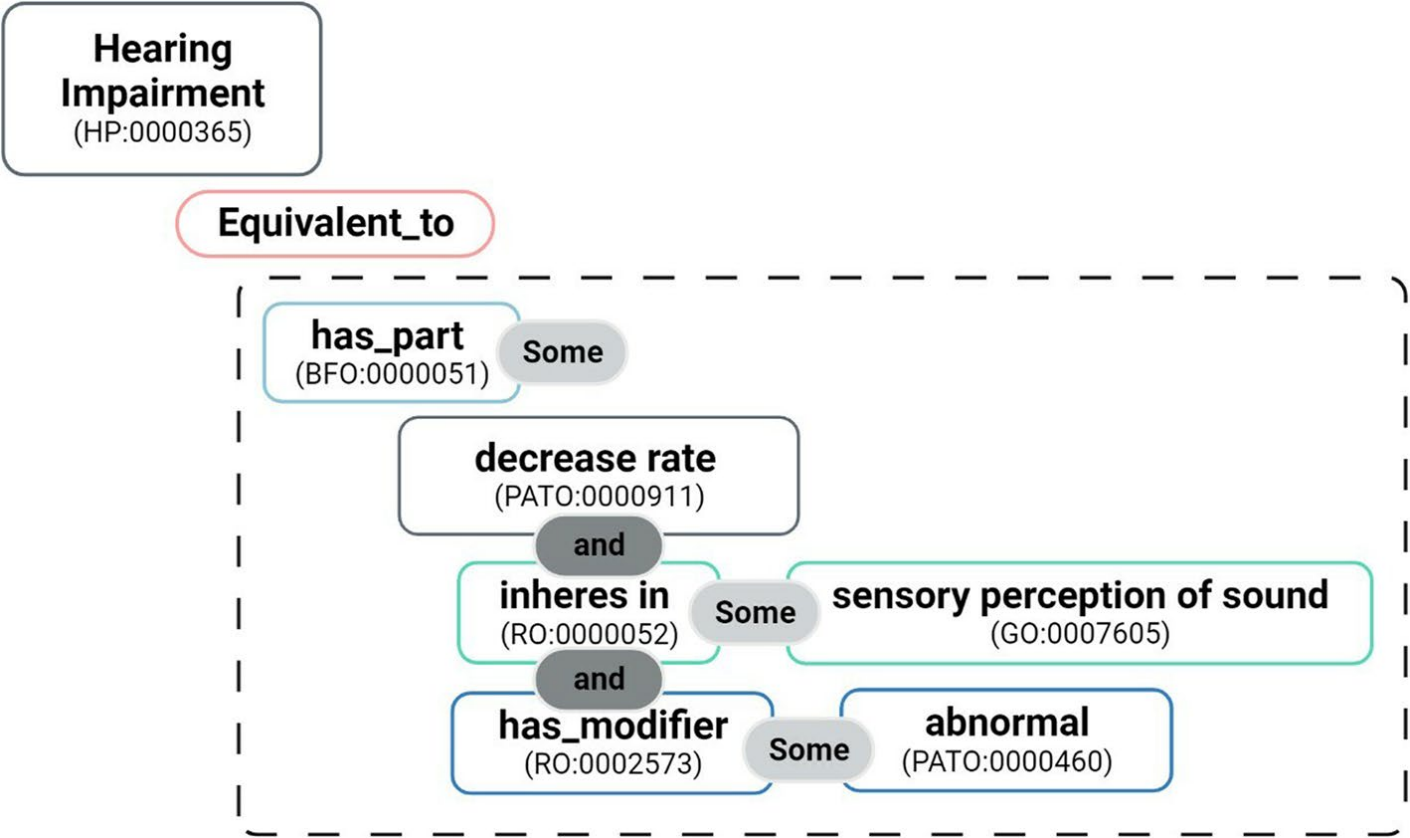
Example of a logical definition of the class Human Phenotype ontology class for “Hearing impairment” (HP:0000365): ’Hearing impairment’ EquivalentTo ’has part’ some (’decreased rate’ and (’inheres in’ some ’sensory perception of sound’) and (’has modifier’ some ’abnormal’))

The HP also includes logical definitions that provide a definition of its classes in terms of a composition of classes from different ontologies with complex semantic relations, facilitating interoperability and data integration [25]. Logical definitions can be explored to bridge domains and contextualize relations between different entities, such as genes and diseases. An example of a logical definition is the one that states that the Human Phenotype ontology class for “Hearing impairment” (HP:0000365) is equivalent to a restriction that involves four other ontologies, as depicted in Fig. 3.

To uncover additional links between HP and GO, we employed AML-Compound^2^ [26] a variant of the AgreementMakerLight ontology matching system that is able to retrieve relations between ontology classes [27, 28]. Using an empirically determined threshold of 0.8, we found 494 mappings, where 37 were identical to the existing logical definitions.

### Knowledge graph integration

In simple terms, a knowledge graph is composed by entities, their relations and an ontology that describes their domain [29]. We built different knowledge graphs composed by different sets of ontologies and types of semantic links between them. In these graphs, entities are not instances of classes of the ontologies in the graph (the ontologies do not describe what genes and diseases are), but are connected to the ontology classes that describe their different aspects.

**Fig. 4:**
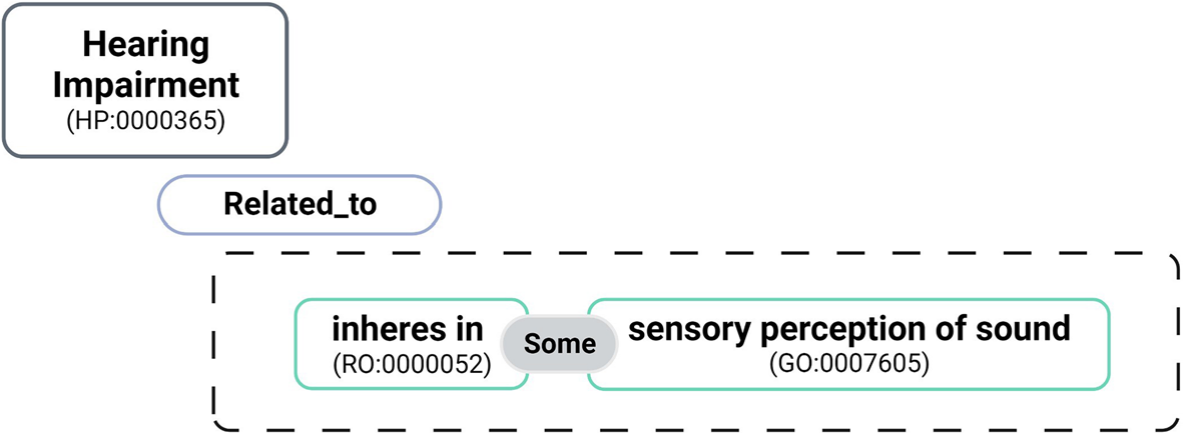
Example of a Logical definition simplified with a more direct relation between two classes. The HP term for “Hearing impairment” (HP:0000365) is related to a restriction that involves the GO term “Sensory perception of sound” (GO:0007605)

To evaluate the impact that knowledge graph semantic richness has on gene-disease association prediction we created different knowledge graphs:

(i) **HPf:** composed by the full version of the HP ontology and annotations for genes and diseases;
(ii) **HPf+GO:** composed by the full version of the HP ontology merged with GO using a virtual root, and including HP annotations (for genes and diseases) and GO annotations (for genes);
(iii) **HPs+GO+LD:** composed by HP without logical definitions merged with GO using a virtual root, HP annotations (for genes and diseases) and GO annotations (for genes) and logical definitions for HP classes that reference GO;
(iv) **HPs+GO+Map:** composed by HP without logical definitions merged with GO using a virtual root, HP annotations (for genes and diseases) and GO annotations (for genes) and mappings between HP classes and GO classes;
(v) **HPs+GO+LD+Map:** the union of *HPs+GO+LD* and *HPs+GO+Map*;

*HPf* represents the baseline, where a single ontology is used. *HPf+GO*, represents an enriched knowledge graph, with two ontologies being used and all logical definitions present in HP. We created other three variants based on a streamlined version of *HPf+GO* where all logical definitions present in HPf were removed which is strategically enriched with only logical definitions and/or mappings with the GO to produce the final three knowledge graphs. Regarding these, to simplify the graph embeddings approach, as seen in the example of Fig. 4, the existing logical definitions and mappings are simplified to a more direct relation between the HP class and GO class through an equivalent class statement. This allows the extraction of a single triple that includes classes from each ontology to power triple-based approaches, and shorter paths linking the ontologies to support random-walk based knowledge graph embeddings methods. The simplification is important given each original logical definition has restrictions with four other ontologies when we only want to gather relations between HP and one of them specifically. Table 1 summarizes relevant statistics about the ontologies for each knowledge graph as well as knowledge graphs without annotations to specific branches created for further studies.

**Table 1:** Statistics for each ontology and knowledge graph regarding classes, annotations, logical definitions and mappings

	**HP**	**HP(-F)**	**HP(-PA)**	**HP(-MI)**	**HP(-CC)**	**HP(-CM)**	**HP(Only PA)**
Classes	15340						
Gene Annotations	136068	1360067	5988	131960	136050	135696	130080
Disease Annotations	40583	40583	2593	38642	40572	40471	37990
	**GO**	**GO(-BP)**	**GO(-CC)**	**GO(-MF)**	**GO(OnlyBP)**		
Classes	44117						
Gene Annotations	76161	33482	57938	60846	42651		
	**HPf**	**HPs+GO+LD**	**HPs+GO+Map**	**HPs+GO+LD+Map**			
LDs or Mappings	3203	350	494	844			

HP version date October 2020; GO version date December 2020. HP branches: Frequency (*F*); Clinical Course (*CC*); Clinical Modifier (*CM*); Mode of Inheritance (*MI*); Phenotypic Abnormality (*PA*). GO branches: Biological Process (*BP*); Cellular Component (*CC*); Molecular Function (*MF*)

### Knowledge graph embeddings and representation

Knowledge graph embeddings were used to learn feature vectors for entities in each knowledge graph and create representations of each gene and disease, covering four types of popular knowledge graph embeddings methods: Translational Distance (TransE [30]), Geometric (HAKE [31]); Semantic Matching (DistMult [32]); and Path-based (RF2Vec [33], OWL2Vec* [34] and OPA2Vec [14]). Every method generated embeddings with 200 dimensions (Table 1 in Additional file).

Our focus on path-based methods is guided by the intuition that path-based methods are better suited to capture long-range relations. This aspect is relevant in our case, where there are no relations between instances of the graph, so to uncover the relations between genes and diseases, the ontology graph must be explored, making it necessary to capture relations at a greater distance. Moreover, OPA2Vec also explores embeddings of the textual component of the ontologies, which is typically rich in biomedical ontologies, with the HP and GO being no exception.

After the knowledge graph embeddings methods, each gene-disease pair corresponds to two vectors, $f_i(g)$ and $f_i(d)$, associated with a gene and a disease, respectively. We defined a binary operator over the corresponding feature vectors g and d in order to generate a joint representation $r(g,d)$ such that r : $V \times V \longrightarrow \mathbb {R}^{d'}$ where d’ is the representation size for the pair $(g,d)$. Several choices for the binary operator were considered from a set of commonly employed operators with knowledge graph embeddings [35]. The chosen operators are summarized in Table 2.

**Table 2:** Choice of binary operators

Operator	Definition
Concatenation	$f_{i}(g) + g_{i}(d)$
Average	$\frac{f_{i}(g) + g_{i}(d)}{2}$
Hadamard	$f_{i}(g) \times g_{i}(d)$
Weighted-L1	$|f_{i}(g) - g_{i}(d)|$
Weighted-L2	$|f_{i}(g) - g_{i}(d)|^2$

### Gene-disease prediction

The knowledge graph embeddings were used to support prediction using two different approaches: machine learning (with Random Forest (RF) [36], eXtreme Gradient Boosting (XGB) [37]) and similarity-based (with cosine similarity and learned threshold using grid search). A grid search was also employed to obtain optimal parameters for the machine learning algorithms (Table 2 in Additional file).

We performed a stratified ten-fold cross-validation and, for each fold, the Weighted Average of F-measures (WAF) of classifications were assessed and reported in the form of a median. Also, the same folds were used throughout all experiments, including the baseline presented in the following section.

## Results and discussion

Our main experiment is a comparative evaluation using knowledge graphs with different levels of semantic richness resulting from one or more ontologies, and the use of LDs and mappings. Additional experiments focused on different ablations studies that removed gene annotations to HP, or gene and disease annotations related to specific annotations of GO and HP.

### Vector combination approaches for embeddings

One of the challenges in achieving a rich semantic representation of genes and diseases when using knowledge graph embeddings is to define a suitable approach to combine the gene and disease vectors.

**Fig. 5:**
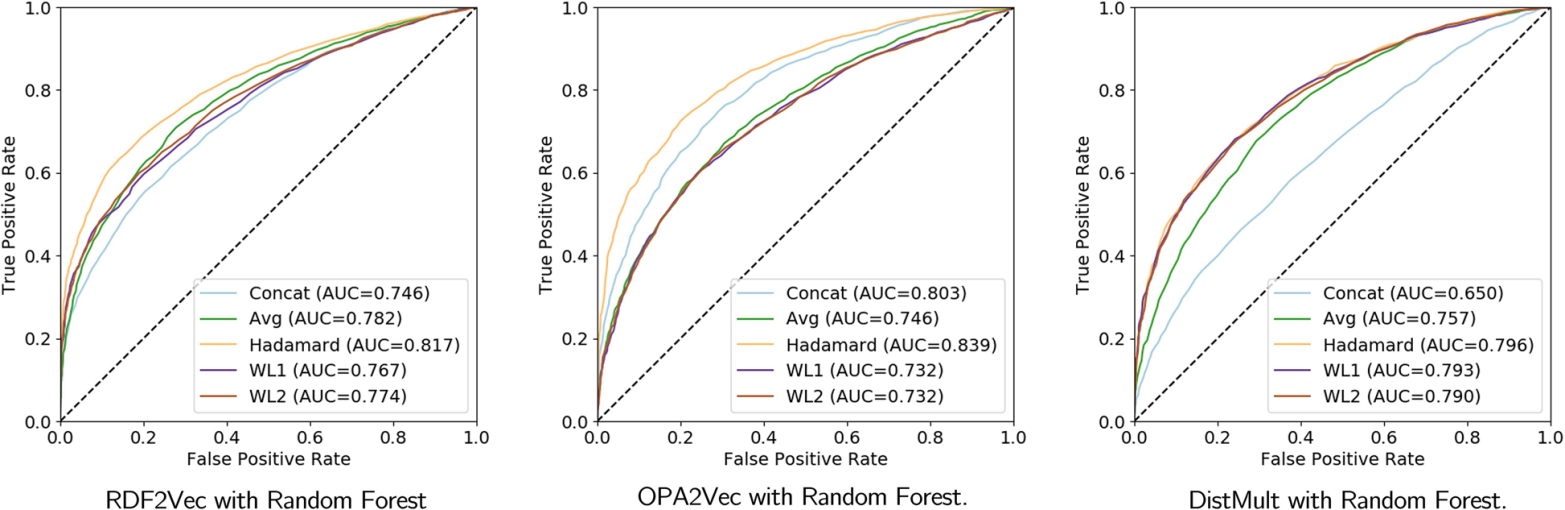
ROC curves and AUC values obtained for different vector operators with RF classifier for the **HP-simple + LD + GO**

Initial experiments with a stratified 70% training and 30% testing split compared the five chosen vector operations with AUC-ROC evaluated using the three best knowledge graph embeddings methods (RDF2Vec, OPA2Vec, and DistMult) coupled with Random Forest classifier (one of the best-performing machine learning algorithms) using the richest knowledge graph (**HP-simple + LD + GO**). The results are summarized in Fig. 5.

The Hadamard operator outperforms other operators when using RDF2Vec, OPA2Vec, and TransE, whereas Concatenation works best with OWL2Vec* and DistMult. Overall, Hadamard and Concatenation are the top two performing combination approaches, with Hadamard achieving the best prediction results when combined with OPA2Vec and Random Forest or XGB. While Hadamard, Average, Weighted-L1, and Weighted-L2 all produce vectors of the same size (200), Concatenation produces double-sized vectors (400). This impacts the training time of the machine learning algorithms. Going forward, all reported experiments employ the Hadamard operator.

### Impact of semantic richness of the knowledge graphs

Table 3 illustrates the impact of employing knowledge graphs with varying degrees of semantic richness with different embedding methods^3^

**Table 3:** Median WAF scores for the combinations of knowledge graph embeddingss with Cosine similarity, RF or XGB for the different knowledge graphs using the Hadamard operator. Best result for each knowledge graph embeddings and machine learning algorithm or CS is bold. Results that are statistically significantly different when compared to HPf are underlined

		RDF2Vec	OPA2Vec	OWL2Vec*	DistMult
**CS**	HPf	0.687	0.671	0.664	0.699
	HPf+GO	0.682	0.670	0.660	0.678
	HPs+GO+LD	**0.689**	**0.677**	0.656	**0.701**
	HPs+GO+Map	0.682	0.667	0.668	0.693
	HPs+GO+LD+Map	0.681	0.676	**0.669**	0.695
**RF**	HPf	0.737	0.756	0.690	0.727
	HPf+GO	***0.753***	0.761	0.696	0.717
	HPs+GO+LD	0.749	0.770	***0.716***	**0.729**
	HPs+GO+Map	0.745	0.771	0.703	0.728
	HPs+GO+LD+Map	0.742	***0.775***	0.711	0.721
**XGB**	HPf	0.732	0.748	0.689	0.728
	HPf+GO	***0.743***	0.758	0.690	0.716
	HPs+GO+LD	0.737	***0.768***	***0.706***	**0.734**
	HPs+GO+Map	0.735	0.765	0.698	0.727
	HPs+GO+LD+Map	0.733	0.765	0.702	0.726

Predictions made with machine learning algorithms achieve better results than cosine similarity. This is unsurprising since reducing the representation of a gene-disease association to a similarity score may be too limiting. Instead, a model learned on multi-dimensional representations is much better at capturing the complexity of the associations.

We can also observe performance differences between knowledge graph embeddings methods. OPA2Vec achieves the best results, with a maximum performance of 0.775 in WAF, followed by RDF2Vec with 0.753. DistMult and OWL2Vec* lag behind with 0.734 and 0.715, respectively.

Multiple factors can explain the better performance of OPA2Vec: it uses asserted and inferred logical axioms in ontologies by using a reasoner; it combines them with vector representations for the lexical component of the ontologies learned over PubMed abstracts using the word2vec model. A clear difference between OPA2Vec and RDF2Vec is the use of rich OWL axioms and word embeddings, which may explain the observed differences. Biomedical ontologies are rich in synonyms, and exploring their similarities in the context of scientific literature can be immensely informative. In other words, this algorithm shows better results because it is better tailored to the specifics of bio-ontologies. Path-based methods appear to be better performers that DistMult, TransE, and HAKE, however OWL2Vec* presents worse results compared to RDF2Vec and OPA2Vec. OWL2Vec* is based on a deeper exploration of OWL axioms which counterintuitively does not improve performance, possibly by introducing noise into the representations. All embedding methods employed receive literals and deal with them differently.

Curiously, knowledge graph embeddings methods show different behaviours depending on the knowledge graph they are applied to. For RDF2Vec, performance is significantly improved over the baseline *HPf* when using *HPf+GO*, but this is not the case for the other knowledge graph embeddings methods. A possible reason behind this is that when a knowledge graph with richer semantics is processed by methods that can explore them, it results in entity vectors that capture many different aspects that may not be relevant for gene-disease association prediction. Another motive could be the proximity in the graph between the HP class declaration and the related GO class. Logical definitions can be quite complex and include many different entities from different ontologies as well as semantic constructs (Fig. 3). In triple oriented methods, such as OPA2Vec and DistMult, the relation between the HP class and the GO class is not directly encoded at the triple-level, and it needs to be learned by jointly training on all triples. In random-walk based methods, such as RDF2Vec, paths linking both classes can be found, making the relation more explicit.

To delve deeper into this issue, the logical definitions declared in the HP ontology were analyzed, and a total of 3203 definitions were identified, but only around 10% of those (350) are related to the Gene Ontology. This motivated the creation of another knowledge graph, *HPs+GO+LD* that addresses both challenges: it only includes logical definitions with GO (potentially removing noise), and it establishes direct links between HP and GO classes (making the relation more explicit in the graph). We also created two more variants *HPs+GO+Map* and *HPs+GO+LD+Map* where mappings between HP and GO found through ontology matching are added to the knowledge graph. When using the three *HPs+GO* variants, both OPA2Vec and OWL2Vec* show significant improvements over the baseline, but DistMult performance is never significantly improved over the baseline regardless of the knowledge graph employed.

**Fig. 6:**
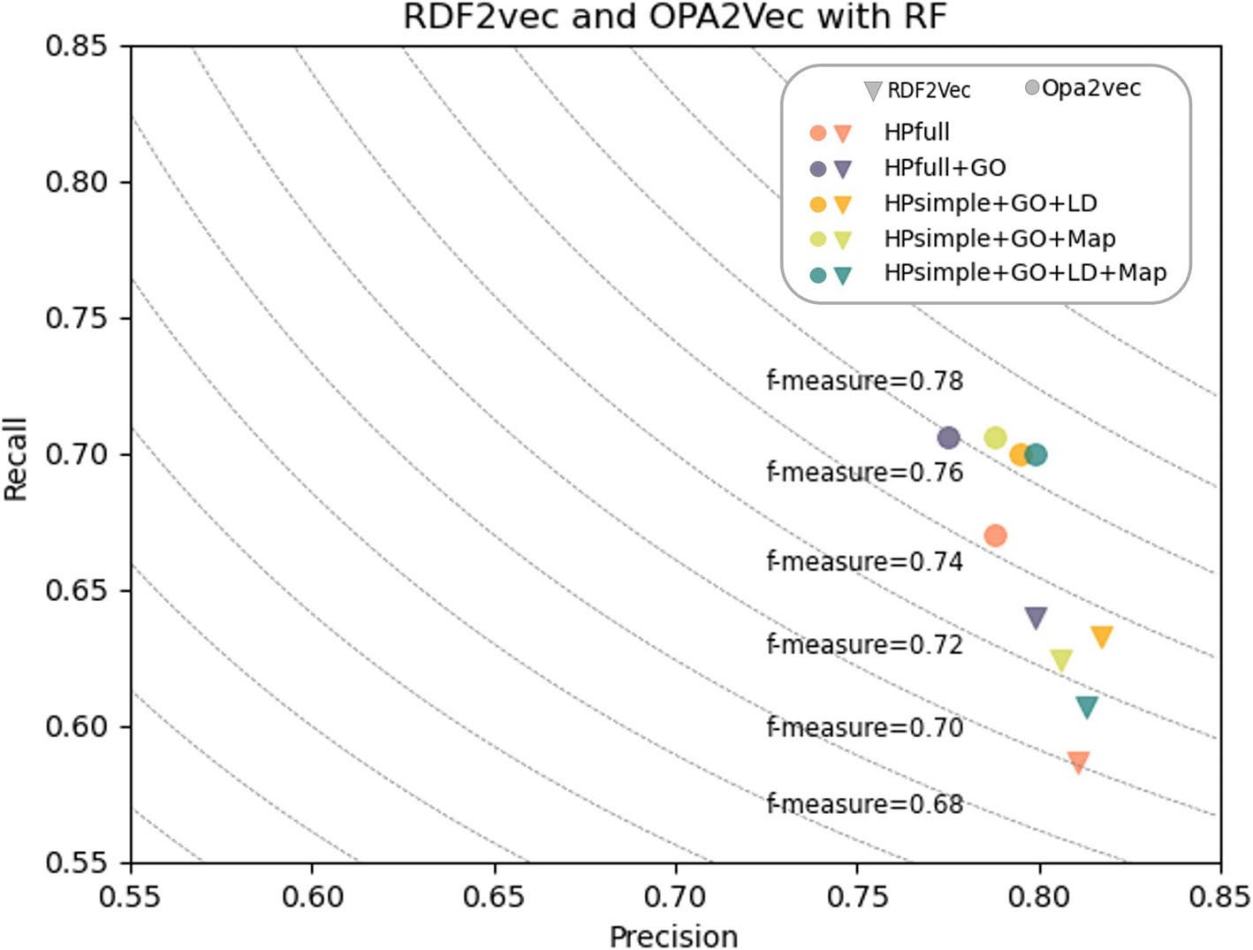
Recall-Precision diagram including f-measure values as height-lines. The diagram uses all knowledge graphs for OPA2Vec and RDF2Vec with RF using a 70-30 split

To better understand the impact of semantic richness, we compared precision and recall values for the five knowledge graphs using OPA2Vec and RDF2Vec embeddings combined with Hadamard operator and a Random Forest model (Fig. 6). In general, for both OPA2Vec and RDF2Vec performance increases with semantic richness, with *HPf* as the knowledge graph with lowest performance in both methods. In both methods, the greater recall gains are seen with *HPf+GO*, but with some precision being sacrificed. Precision is overall improved when using the *HPs+GO* variants, but with greater impacts on precision for RDF2Vec.

Overall, both RDF2Vec, OPA2Vec and OWL2Vec* are able to produce richer semantic representations when given richer knowledge graphs, which in turn improve the prediction of gene-disease predictions.

### Ablation studies

We performed two types of ablation studies to study the impact that a richer ontological layer can have on missing data: (1) removal of the gene annotations using HP; (2) removal of gene and disease annotations of specific branches of the ontologies.

The predictive performance is considerably impacted by the removal of HP gene annotations (Table 4). However, OPA2Vec is still able to achieve WAF values above 0.7. This prediction scenario is perhaps the most realistic one, where knowledge about the phenotype caused by genes is still not known, but disease phenotype and gene function are.

**Table 4:** Median WAF scores for the HP gene annotation ablation study. Best result for each knowledge graph embeddings approach and machine learning algorithm or CS is bold

		RDF2Vec	OPA2Vec	OWL2Vec*	DistMult
**CS**	HPd-f+GO	0.484	**0.532**	0.493	0.499
	HPd-s+GO+LD	0.503	0.525	**0.502**	0.497
	HPd-s+GO+Map	0.463	0.526	0.495	0.500
	HPd-s+GO+LD+Map	**0.506**	0.525	**0.502**	**0.502**
**RF**	HPd-f+GO	**0.664**	0.689	0.597	0.551
	HPd-s+GO+LD	0.657	**0.703**	0.604	**0.576**
	HPd-s+GO+Map	0.659	0.700	**0.617**	0.568
	HPd-s+GO+LD+Map	0.663	0.702	**0.617**	**0.576**
**XGB**	HPd-f+GO	**0.635**	0.672	0.571	0.547
	HPd-s+GO+LD	0.634	0.683	0.587	0.565
	HPd-s+GO+Map	0.632	0.679	0.588	**0.570**
	HPd-s+GO+LD+Map	0.631	0.684	**0.595**	0.569

Table 5 presents the ontology branch annotations ablation studies, taking as a baseline **HPs+GO+LD** and using RDF2Vec and OPA2Vec with the Hadamard operator for RF and XGB.

**Table 5:** Median WAF scores for the ontology ablation studies. Comparison of the best knowledge graph embeddings methods RDF2Vec and OPA2Vec with Random Forest or XGB for the knowledge graph HPs+GO+LD. Results that are statistically significantly different when compared to HPs+GO+LD are underlined. Best results in bold

		Random Forest		XGB	
		RDF2vec	OPA2vec	RDF2vec	OPA2vec
	HPs+GO+LD	**0.749**	**0.770**	0.737	**0.768**
**GO Ablation**	HPs+GO(-BP)+LD	0.747	0.763	0.740	0.759
	HPs+GO(-CC)+LD	0.744	0.767	**0.765**	0.765
	HPs+GO(-MF)+LD	**0.749**	0.770	0.748	0.763
	HPs+GO(Only BP)+LD	0.742	0.769	0.730	0.767
**HP Ablation**	HPs+GO(-F)+LD	0.720	0.740	0.713	0.734
	HPs+GO(-PA)+LD	0.553	0.564	0.562	0.567
	HPs+GO(-MI)+LD	0.723	0.738	0.716	0.738
	HPs+GO(-CC)+LD	0.712	0.733	0.713	0.731
	HPs+GO(-CM)+LD	0.731	0.736	0.714	0.733
	HPs+GO(Only PA)+LD	0.743	0.735	0.742	0.734

The GO ablation studies show that in most cases, the removal of annotations of a single branch, or considering just *biological process* (BP) annotations has little to no impact on prediction. The exception is the removal of *cellular component* (CC) annotations which positively impacts predictions made by XGB coupled with RDF2Vec. It appears that the removal of any branch of the GO ontology is at least partly compensated by the inclusion of logical definitions.

The HP ablation studies show that the annotations removal of any branch significantly lowers performance, with the removal of *phenotypic abnormality* annotations producing the largest decrease. When considering only *phenotypic abnormality* annotations, performance is less affected. This indicates that HP annotations of any branch are essential for the prediction and cannot be compensated by logical definitions.

### Scalability study

As knowledge graphs grow larger and more complex, ensuring the knowledge graph embeddings can handle it efficiently becomes increasingly important. We investigate the scalability of the knowledge graph embedding methods by analyzing their runtime when applied to differently sized knowledge graphs.

**Fig. 7:**
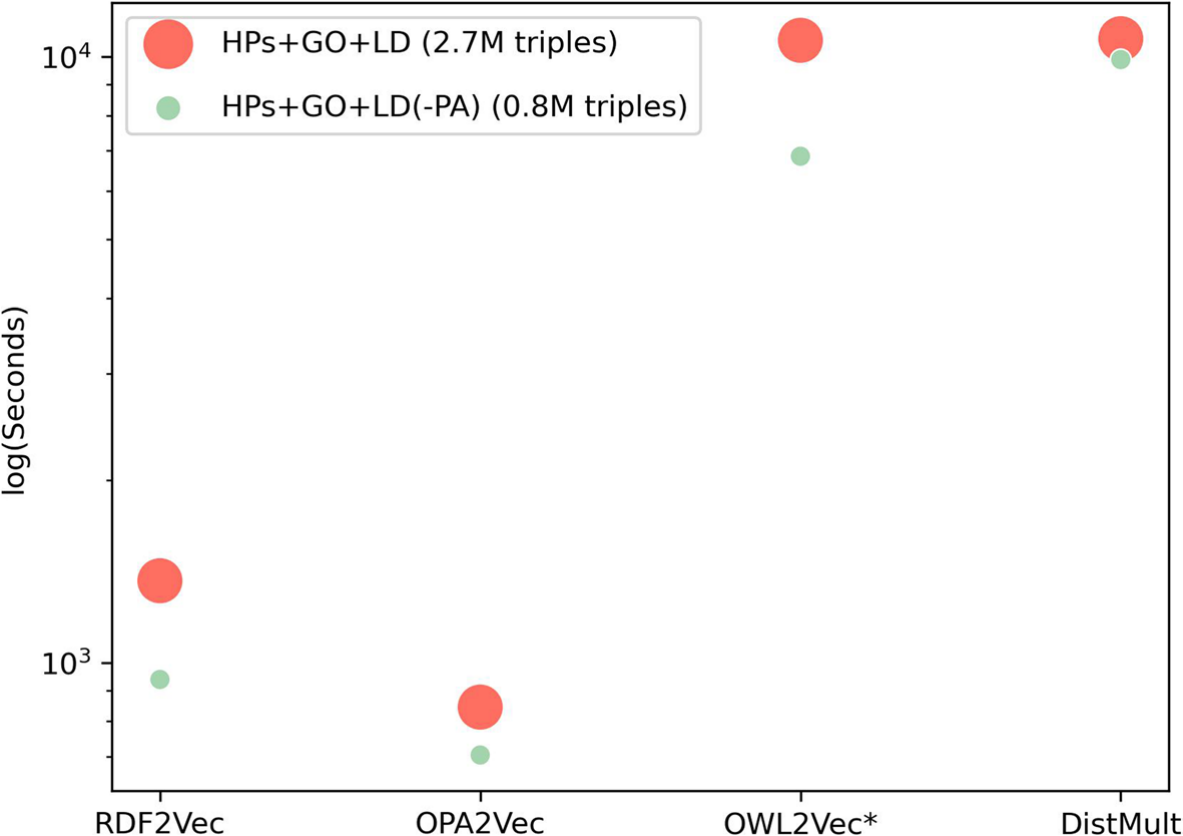
Computational time for each embedding method with two knowledge graphs where the smallest size corresponds to removing the main branch of the human phenotype ontology (Phenotypic abnormality)

Figure 7 shows the results of the computational time for the best embedding methods with two knowledge graphs where the smallest size corresponds to removing the main branch of the human phenotype ontology (Phenotypic abnormality). We can see by the results that RDF2Vec and OPA2Vec are the promptest methods, while OWL2Vec and DistMult are slower. We also can observe that the increase in the size of the knowledge graph is proportional to the increase of the computational time.

When comparing different embedding methods, we must consider whether they utilize path-based strategies (random walks) or access triples. For OPA2Vec, TransE, and DistMult, embeddings were generated using triples. In contrast, RDF2Vec and OWL2Vec utilized random walks for generating embeddings. Specifically, 500 random walks were generated for each knowledge graph for RDF2Vec and OWL2Vec. Furthermore, the entities used for learning the embeddings varied among the different methods. RDF2Vec and OWL2Vec only generated embeddings for the entities asked. OPA2Vec, TransE, and DistMult generated embeddings for all entities in the knowledge graph.

## Conclusions

Deciphering the links between genes and diseases is a crucial area of research. Computational approaches present themselves as an answer to the data deluge in the life sciences, and ontologies and knowledge graphs have become increasingly crucial to support data-intensive applications in biology, in particular, the prediction and prioritization of gene-disease associations.

We proposed a novel approach to predict gene-disease associations using rich semantic representations based on knowledge graph embeddings over multiple ontologies, in this case, the Human Phenotype Ontology and the Gene Ontology. We investigated different approaches to build a shared rich semantic representation for genes and diseases exploring both logical definitions and compound ontology matching, and how these different approaches influence the performance of representative knowledge graph embeddings methods. A benchmark dataset without potential data leakage was created to support evaluation, ensuring its appropriateness for gene-disease prediction.

Our experiments showed that considering richer knowledge graphs, composed by more than one ontology and with rich links between them significantly improve gene-disease prediction based on knowledge graph embeddings. Interestingly, different knowledge graph embeddings methods benefit more from distinct types of semantic richness. While the performance of RDF2Vec improves more when considering the most complete version of HP with all logical definitions integrated with GO, OPA2Vec and OWL2Vec* achieve their best performance when considering a streamlined version of HP with direct links to GO generated by exploring the logical definitions. It is likely that the reliance of OPA2Vec and OWL2Vec* on lexical information results in the introduction of noise when considering the full spectra of logical definitions. We also determined that in the absence of logical definitions, strategies for compound ontology alignment can be employed to establish rich links across ontologies that cover different domains. Despite this, it is important to note that graph convolutional networks (GCNs) and other graph neural network-based methods were not considered in this study, as the focus was on exploring the potential of knowledge graph embeddings across multiple ontologies and this type of methods are not yet tailored for the semantic richness of the KGs. Furthermore, Graph Neural Networks (GNNs) algorithms rely on message passing and require node features, which are the messages passed through the edges. However, ontologies are often not rich in node properties, with only labels available in most cases. Moreover, textual node properties are not easily represented in a vectorial format, which is the typical approach used by GNNs. Therefore, adapting GNNs to such problems can be challenging.

This work demonstrated the potential for knowledge graph embeddings across multiple and interconnected biomedical ontologies to support gene-disease prediction. All software is freely available and the approach can be easily generalized to consider other ontologies (for instance the Disease Ontology or the ChEBI ontology) and to solve different tasks where multiple perspectives over the data can be beneficial (e.g., protein function prediction, protein-protein interaction prediction, or patient-disease prediction, etc.).

### Supplementary Information

**Additional file 1.**

## Data Availability

All data and code are available at: https://github.com/liseda-lab/KGE_Predictions_GD.

## Abbreviations

AUC-ROC: Area under the receiver operating characteristic curve
GO: Gene ontology
HP: Human phenotype ontology
KG: Knowledge graph
KGE: Knowledge graph embeddings
LD: Logical definitions
OMIM: Online mendelian inheritance in man
OWL: Web ontology language
RF: Random Forest
WAF: Weighted average of f-measures
XGB: eXtreme gradient boosting

## Footnotes

1 Machine specifications: Ncores: 16; Disk: SSD 1TB + 4TB HDD; RAM: 64 GB

2 https://github.com/AgreementMakerLight/AML-Compound

3 TransE and HAKE underperformed (Table 3 in Additional file) and predictive approaches.
