# Supplementary material for: Multi-domain knowledge graph embeddings for gene-disease association prediction
Source: J Biomed Semantics. 2023 Aug 14;14:11. doi: 10.1186/s13326-023-00291-x (PMC10426189; doi:10.1186/s13326-023-00291-x)
Supplement: Supplementary file 1 — Additional file 1. [file 13326_2023_291_MOESM1_ESM.pdf]

Additional Files

Table 1: Parameters for the knowledge graph embeddings.

	Parameters	Literals
**RDF2Vec /OWL2Vec**	nr_walks = 500, walks_depth= 8, walker=WeisfeilerLehmanWalker, sampler=UniformSampler<br>Word2vec default parameters: sentences=None, corpus file=None, alpha=0.025,<br>window=5, min count=5, max vocab size=None, sample=0.001,<br>seed=1, workers=3, min alpha=0.0001, sg=0, hs=0, negative=5,<br>ns exponent=0.75, hashfxn=, epochs=5,<br>null word=0, trim rule=None, sorted vocab=1, batch words=10000,<br>compute loss=False, callbacks=(), comment=None, max final vocab=None,<br>shrink windows=True	Yes
**OPA2Vec**	annotations [metadata annotations]: All annotation properties.<br>pretrained [pre-trained model]: Default pre-trained model from http://bio2vec.net/data/pubmed model/<br>reasoner [reasoner]: Elk<br>debug [debug]: set to no, in which case no intermediate files are kept once the program exits.	Yes
**OpenKE (DistMult/TransE/TransH)**	work threads(4), train times(500), nbatches(100), alpha(0.001), margin(1.0),<br>bern(0), dimension(20), ent neg rate(1),rel neg rate(0), opt method("SGD")	Yes

Table 2: Grid-Search parameters for the machine learning algorithms.

**Algorithm**	**Parameters**	**Values**
RF	maximum depth	2, 4, 6, None
	nr of estimatores	50, 100, 200
XGB	maximum depth	2, 4, 6
	nr of estimatores	50, 100, 200
	learning_rate	0.1, 0.01, 0.001
MLP	hidden layer sizes	(50, 50, 50), (50, 100, 50), (100,)
	activation	tanh, relu
	solver	sgd, adam
	alpha	0.0001, 0.05
	learning_rate	constant, adaptive

Table 3: Median WAF scores for TransE and HAKE with Cosine similarity, RF or XGB for the different knowledge graphs using the Hadamard operator. Best result for each knowledge graph embedding method and machine learning algorithm or CS is bold.

		**TransE**	**HAKE**
**CS**	HPf	0.522	**0.531**
	HPf+GO	0.513	0.512
	HPs+GO+LD	**0.516**	0.506
	HPs+GO+Map	0.511	0.514
	HPs+GO+LD+Map	0.512	0.521
**RF**	HPf	**0.498**	**0.681**
	HPf+GO	0.471	0.592
	HPs+GO+LD	0.487	0.616
	HPs+GO+Map	0.496	0.610
	HPs+GO+LD+Map	0.496	0.608
**XGB**	HPf	0.509	**0.691**
	HPf+GO	0.493	0.617
	HPs+GO+LD	**0.515**	0.633
	HPs+GO+Map	0.509	0.641
	HPs+GO+LD+Map	0.509	0.649
